# Supplementary material for: Content-rich biological network constructed by mining PubMed abstracts
Source: BMC Bioinformatics. 2004 Oct 8;5:147. doi: 10.1186/1471-2105-5-147 (PMC528731; doi:10.1186/1471-2105-5-147)
Supplement: Additional File 2 — The original results of the above study (non-essential files are deleted to keep the file size under the limit set by BMC bioinformatics). [file 1471-2105-5-147-S2.bz2 › chilibotAdditionalFile2/dip10/484ID8702798E10575/html/IL8RA_PPBP.html]

 


 **IL8RA** and **PPBP** 
  
Found 25 abstracts in PubMed, retrieved 10.  
 

 What does Google say? 
 PDF only 
| .edu only 

---

**Interactive relationship** (e.g. stimulation, inhibition, etc)

**Neutral relationship**- Immunoprecipitation and Western blot analyses of surface expressed receptors covalently linked to IL 8 or NAP 2  [ **PPBP** ]  as well as in their unloaded state revealed the occurrence of a single CXCR  [ **IL8RA** ]  2 variant with an apparent size of 56 kDa.  Ref: 10878382 J Immunol, 2000
- Similar to other members of this group, such as IL 8, NAP 2  [ **PPBP** ]  activates chemotaxis and degranulation in neutrophils polymorphonuclear PMN through chemokine receptors CXCR 1  [ **IL8RA** ]  and CXCR  [ **IL8RA** ]  2.  Ref: 11049972 Blood, 2000
- CXCR1  [ **IL8RA** ]  and CXCR2 mediate migratory activities in response to IL 8 and other ELR CXC chemokines e.g., GCP 2 and NAP 2  [ **PPBP** ] .  Ref: 12627953 Biochemistry, 2003
- The G protein coupled CXC chemokine receptor CXCR  [ **IL8RA** ]  2 mediates activation of neutrophil effector functions in response to multiple ligands, including IL 8 and neutrophil activating peptide 2 NAP 2  [ **PPBP** ] .  Ref: 10878382 J Immunol, 2000
- After phagocytosis of opsonized yeast, the expression of CXCR1  [ **IL8RA** ]  and CXCR2 was substantially down regulated and was accompanied by reduced Ca responses to corresponding ligands, IL 8 and neutrophil activating peptide 2 NAP 2  [ **PPBP** ] .  Ref: 12239185 Blood, 2002
- These receptors did not participate in ligand induced receptor trafficking, while surface expressed CXCR  [ **IL8RA** ]  2 was internalized and re expressed following stimulation with NAP 2  [ **PPBP** ] .  Ref: 10878382 J Immunol, 2000

**Non-interactive relationship** (e.g. studied together, co-existance, homology, etc.)

- By enzymatic removal of one 9 kDa carbohydrate moiety in surface expressed CXCR  [ **IL8RA** ]  2 we can show that neither NAP 2  [ **PPBP** ]  induced trafficking nor signaling of the receptor is dependent on its full glycosylation.  Ref: 10878382 J Immunol, 2000
- Correspondingly, we found PBP  [ **PPBP** ]  and, less potently, CTAP III to inhibit CXCR  [ **IL8RA** ]  2 but not CXCR 1  [ **IL8RA** ]  dependent chemotaxis of neutrophils toward NAP 2  [ **PPBP** ] .  Ref: 11049972 Blood, 2000
- Transendothelial migration of neutrophils in response to CXCL7  [ **PPBP** ]  or IL 8 CXCL8 was subject to modulation by CTAP III, but not CXCL4, as seen by drastic desensitization of the migratory response of neutrophils pre exposed to CTAP III, which was paralleled by selective down modulation of CXCR  [ **IL8RA** ]  2.  Ref: 12193731 J Immunol, 2002
- Thus, proteolytic processing of inactive precursors plays a decisive role in whether the beta TG  [ **PPBP** ]  proteins will act as stimulatory or inhibitory agents in neutrophil activation via the G protein coupled receptors CXCR 1  [ **IL8RA** ]  and 2.  Ref: 11138777 Immunol Rev, 2000
- This was evident from experiments in which inhibition of the CTAP III processing protease and simultaneous blockade of the CXCL7  [ **PPBP** ]  high affinity receptor CXCR  [ **IL8RA** ]  2 led to complete abrogation of CTAP III mediated neutrophil adhesion.  Ref: 12193731 J Immunol, 2002
